# Detection and Confirmation of *Naegleria fowleri* in a Primary Amebic Meningoencephalitis Patient Using a Molecular Approach

**DOI:** 10.1155/2024/5514520

**Published:** 2024-11-26

**Authors:** Muhammad Aurongzeb, Muhammad Amer Nazir, Raheela Yasmin, Ammeema Kiran, Raiha Fatima, Rehan Ali, Salman Ahmed Khan, Asad Ul-Haq, Khalid Al-Regaiey, Turki Abualait, Imdad Kaleem, Shahid Bashir

**Affiliations:** ^1^Department of Applied Sciences, FEST, Hamdard University, Karachi 74600, Pakistan; ^2^HITEC-IMS Taxila, National University of Medical Sciences (NUMS), Rawalpindi, Pakistan; ^3^Department of Biosciences, COMSATS University Islamabad (CUI), Islamabad, Pakistan; ^4^Department of Biosciences, Faculty of Life Sciences, Shaheed Zulfikar Ali Bhutto Institute of Science and Technology (SZABIST), Karachi 75600, Pakistan; ^5^Department of Molecular Medicine, Dow College of Biotechnology, Dow University of Health Sciences, Karachi, Pakistan; ^6^Division of Rheumatology, Department of Internal Medicine, Soonchunhyang University Seoul Hospital, Seoul, Republic of Korea; ^7^Department of Physiology, College of Medicine, King Saud University, Riyadh, Saudi Arabia; ^8^College of Applied Medical Sciences, Imam Abdulrahman Bin Faisal University, Dammam, Eastern Province 34212, Saudi Arabia; ^9^Neuroscience Center, King Fahed Specialist Hospital, Dammam, Saudi Arabia

**Keywords:** amoebic infection, cerebrospinal fluid, Karachi, meningitis, *Naegleria fowleri*, Pakistan, primary amebic meningoencephalitis

## Abstract

The *Naegleria fowleri* amoeba stands as the primary culprit behind primary amebic meningoencephalitis (PAM), presenting a substantial global public health concern. In recent years, over 17 cases of PAM have been reported in Karachi, Pakistan, highlighting its increased prevalence in the country's most densely populated city. This study scrutinized 74 cerebrospinal fluid (CSF) samples collected from meningitis patients across various health facilities in the city. These samples underwent thorough examination employing biochemical, microbial, and cytological methods. Additionally, polymerase chain reaction (PCR) with specific primers targeting the *Naegleria* genus and *N. fowleri* was employed to ascertain the presence of *N. fowleri* in the CSF samples. While biochemical and cytological analyses provided supportive information, they failed to yield a distinct diagnostic pattern. Nevertheless, through direct microscopic observation, cultural growth, and PCR-based analyses, *N. fowleri* was definitively identified in one CSF sample.

## 1. Introduction

Primary amoebic meningoencephalitis (PAM), caused by the amoeba *Naegleria fowleri*, has been reported in various areas of the world, including Pakistan, America, Australia, and East Asian countries [1–3]. Over the past five decades, numerous cases of PAM have been documented, with the first patient diagnosed in Australia in 1965 [4]. In Pakistan, the first case was reported in Karachi in June 2008 [2]. From then until 2023, more than a 100 cases of this infection have been recorded in the country [5–7]. According to the World Health Organization (WHO), *N. fowleri*, which infects the central nervous system (CNS), is the second most prevalent factor behind morbidity and mortality globally [8]. It is classified as a thermophilic free-living amoeba (FLA), from the kingdom Protista, subkingdom Protozoa, phylum Sarcomastigophora (Sarcodina), and the superclass Rhizopodia. It is commonly found in humid soil and freshwater environments worldwide [9]. *N. fowleri* thrives in warm aquatic habitats such as lakes, streams, pools, water reservoirs, and water supplies [2, 9, 10]. The majority of PAM cases are noticed during the summer when temperatures rise between 40°C and 46°C, as *N. fowleri* exhibits increased proliferation in warm weather [11–13]. Young adults and children are more commonly affected, particularly those engaged in water sports, swimming in warm freshwater and direct exposure to tap water [2]. The life cycle of *N. fowleri* involves three distinct morphological stages: cyst, flagellated, and trophozoite stages, with the trophozoites being the causative agents [14, 15]. Infection occurs when people come into contact with contaminated water, allowing the amoeba to enter the body through the nasal cavity. The flagellated amoebae then transform into trophozoite amoebae and attach themselves to the mucosa. They migrate along the nerves, eventually invading the CNS and causing PAM [16–18]. In nonolfactory regions of the nasal cavity, *N. fowleri* does not elicit symptoms such as inflammation, sneezing, or tissue damage [18]. However, upon entering the CNS*, N. fowleri* trophozoites inflict significant brain tissue damage accompanied by inflammation and hemolysis, affecting nerve cells. In terms of sign and symptoms, a significant level of similarity has been reported between the PAM and the bacterial meningitis, making it difficult to differentiate between the two. The delayed diagnosis of PAM contributes to its high mortality rate [2, 19–21]. Consequently, PAM is recognized as a serious global health concern [22]. In this study, we present the findings of our research on the isolation, identification, and detection of *N. fowleri* from patients with PAM in Karachi, Pakistan.

## 2. Materials and Methods

### 2.1. Collection of Cerebrospinal Fluid (CSF) Samples

A total of 74 turbid or slightly turbid CSF samples from suspected meningitis patients were collected from different diagnostic centers and hospitals of Karachi, Pakistan from August 2015 to September 2020.

### 2.2. Biochemical and Microscopic Analysis of CSF Samples

Biochemical and microscopic analysis of CSF samples was conducted as follows to ensure timely processing and accurate measurements. All samples were processed within 2 h of collection. While a fraction of each sample underwent centrifugation at a speed of 2000x*g* for 15 min at room temperature (23–24°C). The CSF supernatant was used to determine glucose and total protein levels. The entire CSF samples were subjected to Sysmix KX 21N hematology analyzer to obtain the total count and differential count of leukocytes. Meanwhile, the pellet obtained from each CSF sample was resuspended in 200 *μ*L of supernatant and allowed to incubate for 30 min at 37°C. Subsequently, direct microscopic examination of the resuspended pellet was performed using a compound microscope with a 40x lens. This was accomplished by placing 100 *μ*L of the resuspended pellet onto a glass slide.

### 2.3. Culture-Based Identification of *N. fowleri*

CSF samples underwent centrifugation as described earlier, and the resulting sediments were gently resuspended in 100 *μ*L of the supernatant. The resuspended material was carefully pipetted onto the mid of the plates of nonnutrient agar (NNA), which were prepared by layering a suspension of *Escherichia coli* ATCC number 25922 in PAGE amoeba saline. The NNA culture was kept at 44°C for a maximum period of 10 days after sealing them by using parafilm [23, 24]. The temperature of 44°C is specifically chosen for the isolation of *N. fowleri*, as only the thermophilic amoeba *N. fowler*i can survive at this temperature [25]. Daily examinations of the NNA plates were conducted for duration of 10 days using a 400x light microscope.

### 2.4. Extraction of Genomic DNA From CSF

The CSF samples underwent centrifugation at a speed of 10,000×*g* for 10 min. The resulting sediments were then utilized for DNA extraction with the QIAamp DNA Mini Kit (Qiagen Inc., USA).

### 2.5. *N. fowleri* Polymerase Chain Reaction (PCR)–Based Detection

PCR amplification was conducted to verify the presence of motile amoeba due to the challenge in selectively identifying *Naegleria* spp. and/or *N. fowleri* through morphology. Due to the difficulty in specifically recognizing *Naegleria* spp. and/or *N. fowleri* based on cellular morphology, PCR amplification was carried out to confirm the presence of motile amoeba. Both positive and negative purulent CSF samples were subjected to the PCR amplification procedure, which was carried out in accordance with previously described techniques [26–29]. We need a total volume of 25 *μ*L to conduct the amplification experiment, which contain 5 *μ*L of DNA from the sample which works as a template, 9.5 *μ*L of double-distilled water, 0.5 *μ*L of each primer (10 *μ*M), and 10 *μ*L of Master Mix (Promega, USA). Three sets of primers were used to find *Naegleria* spp. or *N. fowleri* in the samples are as follows:

Nf-ITS1-F GAACCTGCGTAGGGATCATTT

Nf-ITS2-R TTTCTTTTCCTCCCCTTATTA [30, 31]

NaeglF1925-F GTGCTGAAACCTAGCTATTGTAACTCAGT

NaeglR344-R CACTAGAAAAAGCAAACCTGAAAGG [2]

Primer pairs were used in 40 cycles of amplification reactions following a heat treatment at 95°C for 5 min to achieve protracted denaturation. Each cycle included 3 s of denaturation at 95°C, 30 s at 53°C for annealing, and 30 s of extension at 72°C. A final extension phase was carried out for 5 min at 72°C. Then, a 2% agarose gel was used to observe the amplified result [26].

## 3. Results

### 3.1. Biochemical and Cytological Analysis of CSF Samples

Biochemical and cytological analyses of CSF samples were performed in this work. Overall, 74 CSF samples were included in the research, collected from 40 males (54%) and 34 females (46%). The patients were aged between 2 and 71 years, with a mean of 38.33 ± 10.44. The findings of the CSF samples, including biochemical and cytological data, can be found in Table 1. The analysis of CSF chemistry revealed that all samples had an elevated protein level (> 40 mg/dL), and 83.78% (*n* = 62) of them had a decreased glucose level (< 50 mg/dL). Furthermore, 58.11% (*n* = 43) of the samples had a leukocyte count of > 1000 cells/mm^3^, while 39.1% (*n* = 29) had a leukocyte count between 100 and 1000 cells/mm^3^. The majority of the samples, 78.37% (*n* = 58), exhibited a predominance of neutrophils in the CSF (neutrophil count). Out of the 74 CSF samples collected from patients suspected of having meningitis, only one sample tested positive for *N. fowleri* using PCR analysis. The biochemical and cytological parameters of the PCR-positive sample (*n* = 1) were compared with the median values of the PCR-negative samples (*n* = 73). The results revealed a common pattern in both groups including low glucose concentration, elevated protein concentration, and increased leukocyte count. A consistent decrease in glucose levels was observed, which is characteristic of an infection but is not specific to any particular type while both PCR-positive and PCR-negative samples displayed high protein levels, suggesting inflammation or disruption in the blood–brain barrier. Moreover, a significant increase in leukocytes, particularly neutrophils, was observed, indicative of an inflammatory response. These findings are consistent with observations in other forms of meningitis, making it challenging to distinguish between PAM and other types based solely on these parameters (Table 2).

### 3.2. *N. fowleri* Cultivation on NNA

**Figure 1:**
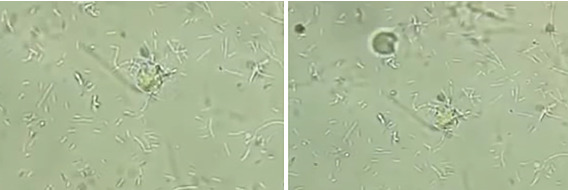
*N. fowleri* trophozoites (400x) from a PAM patient's CSF, cultured on NNA and viewed with a 40x objective.

All the NNA plates (*n* = 74) were examined under a light microscope (OPTIKA, B-382 PLi, Italy) using a 400x magnifying lens on an alternate day basis for 10 days. NNA plates (*n* = 73) that did not exhibit any morphological characteristics of *N. fowleri* within 10 days were regarded as negative and appropriately disposed of through incineration. The trophozoite stage was observed in only one sample (1.35%) on the third day of culturing (Figure 1).

### 3.3. Direct Microscopic Examination of a Patient's CSF Sample

**Figure 2:**
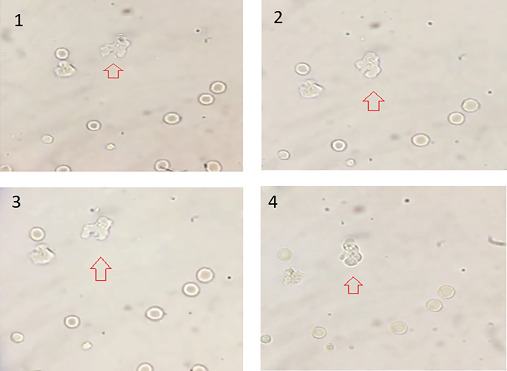
Wet mount of CSF showing *N. fo*wleri (trophozoite form). Arrows depict the trophozoites in various states of movement.

Direct microscopy analysis of freshly prepared CSF smears revealed the presence of active and mobile amoebic cells exhibiting pseudopodia in a single CSF sample (identified as A-27) out of a total of 74 samples examined (Figure 2). The dynamic transformation of cell morphology and the formation of pseudopods in the A-27 sample strongly indicated the presence of amoebic trophozoites. These trophozoites were estimated to be approximately 12–15 *μ*m in size. The observed crawling behavior of the amoeba was characterized by rapid movement, with an approximate speed of 1 *μ*m/s, facilitated by the generation of eruptive pseudopods.

### 3.4. *N. fowleri* Detection by Using PCR (Sample A-27)

**Figure 3:**
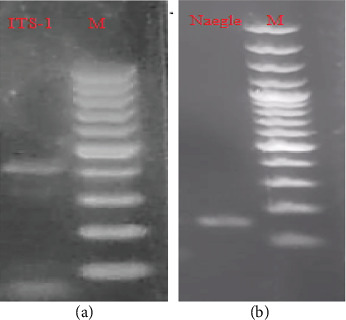
(a, b) Amplicon bands generated from the genomic DNA of *N*. *fowleri* using the specific primers. ITS-1, *Naegleria* spp.; Naegle, *N*. *fowleri*; and M, 100 bp marker.

PCR amplicons obtained using primer pairs Nf-ITS1-F_Nf-ITS1-R and NaeglF1925-F_NaeglR344-R showed approximate lengths of 410 bp (https://http://www.ncbi.nlm.nih.gov/nuccore/[accession number MT726981.1], Figure 3(a)) and 153 bp, respectively (Figure 3(b), the uncropped images of both figures could also be seen in Figure S1 and S2, respectively, in the Supporting Information).

## 4. Discussion

The diagnosis of PAM is challenging because of the nonspecific findings observed in the CSF of PAM patients. These findings, including an increase in neutrophil count, elevated protein levels, and reduced glucose levels, are similar to those seen in acute bacterial meningitis. This similarity often leads to misdiagnosis and, ultimately, the death of the patient. To achieve quick and accurate detection of the disease, it is crucial to directly observe amoebae in a wet mount of fresh CSF. Traditional staining techniques like Gram staining are not effective in this case, as the heat fixation process may lead to the destruction of the organism [32]. However, it is important to note that on a wet mount, *N. fowleri* and motile macrophages are difficult to differentiate, which may result in a false positive diagnosis. There are two ways to obtain a confirmed diagnosis of PAM: (a) by growing amoeba on NNA plates along with *E. coli* and (b) by PCR. In this study, a temperature of 44°C was used for culturing, which can inhibit the growth of other pathogenic species of *Naegleria. Naegleria lovaniensis*, closely related to *N. fowleri*, is found frequently in warm waters and is also thermophilic but nonpathogenic [33]. In different regions, including Pakistan, Taiwan, and Vietnam, PAM has been diagnosed from the CSF of patients using PCR assays based on Naegl primers [2, 34]. Additionally, *N. fowleri* DNA has been amplified using 5.8S ITS (internal transcribed spacer) primers from a CSF sample of a French patient [35–37]. In this study, 74 samples of suspected meningitis patients were screened to isolate, identify, and genotype *N. fowleri.* The samples were subjected to biochemical and cytological analyses, which revealed a general decrease in glucose concentration, an increase in total protein concentration, and an elevated total leukocyte count with a higher percentage of neutrophils. However, there was no statistically significant difference in CSF chemistry and cells between PCR-positive and PCR-negative CSF samples, suggesting that these routine parameters were insufficient for distinguishing *N. fowleri* infection from other forms of meningitis (Table 2). Upon microscopic examination of all the available samples, only one sample (A-27) exhibited motile amoeba, indicating the presence of *Naegleria* species. PCR analysis of all 74 CSF samples confirmed positive results for the same sample (A-27) using ITS, Naegl, primers. The detection of *N. fowleri* in the CSF of this patient supports the diagnosis of PAM in this case. Overall, this study contributes to the understanding of diagnosing PAM using molecular and microscopic methods, emphasizing the importance of direct microscopy and PCR analysis for the accurate detection of *N. fowleri* in suspected PAM cases [38, 39].

## 5. Conclusion

In conclusion, this study underscores the emergence of *N. fowleri* as a significant public health concern in Karachi, Pakistan, marked by a high prevalence of PAM cases. Despite the challenges in differentiating PAM from other forms of meningitis using routine biochemical and cytological assays, advanced molecular techniques, such as PCR, have proven instrumental in confirming the presence of *N. fowleri* in CSF samples. The findings highlight the critical importance of early and accurate diagnosis to enhance patient outcomes and implement appropriate treatment strategies. As outbreaks of *N. fowleri* continue to pose a threat, maintaining vigilance and raising awareness among healthcare professionals in endemic regions is essential to effectively combat this life-threatening infection and safeguard public health.

## Figures and Tables

**Table 1:** Demographic, biochemical, and cytological data of patients (*n* = 74).

**Characteristics**	**Values**
Age (years)	26.04 ± 16.25
Sex	
Male	40 (54.06%)
Female	34 (45.94%)
Glucose (mg/dL)	25.82 ± 20.49
< 50	62 (83.78%)
> 50	12 (16.22%)
Protein (mg/dL)	563.05 ± 546.68
< 40	0 (0.0%)
> 40	74 (100%)
WBCs (total/mm^3^)	2015.99 ± 1877.62
< 100	2 (2.70%)
< 101–1000	29 (39.19%)
> 1000	43 (58.11%)
Neutrophil percentage	70.26 ± 27.72
< 50	16 (21.62%)
> 50	58 (78.38%)
Lymphocyte percentage	30.15 ± 27.49
< 50	58 (78.37%)
> 50	16 (21.62%)

**Table 2:** Comparison of biochemical and cytological factors between PCR-positive and PCR-negative samples.

**Variables**	**PCR-negative**	**PCR-positive**	**Normal values**
	**Median**	**Absolute value**	
Glucose (mg/dL)	24	5	45–80
Protein (mg/dL)	240	240	20–40
Leucocyte (cells/mm^3^)	1500	2260	0–5
Neutrophil percentage	80	75	40–70
Lymphocyte percentage	25	25	20–40

## Data Availability

The data is available on request from the corresponding author.

## Supporting Information

Supporting Information Additional supporting information can be found online in the Supporting Information section. Figure S1: amplicon bands generated from the genomic DNA of *N. fowleri* using the specific primers. ITS-1 and M: 100 bp marker (uncropped image of gel of Figure 3(a) in the manuscript). Figure S2: amplicon bands generated from the genomic DNA of *N. fowleri* using the specific primers. Naegle: *N. fowleri* and M: 100 bp marker (uncropped image of gel of Figure 3(b) in the manuscript).
