# Supplementary material for: Detection and Confirmation of Naegleria fowleri in a Primary Amebic Meningoencephalitis Patient Using a Molecular Approach
Source: J Parasitol Res. 2024 Nov 26;2024:5514520. doi: 10.1155/2024/5514520 (PMC11614516; doi:10.1155/2024/5514520)
Supplement: Supporting Information — Additional supporting information can be found online in the Supporting Information section. Figure S1: amplicon bands generated from the genomic DNA of N. fowleri using the specific primers. ITS-1 and M: 100 bp marker (uncropped image of gel of Figure 3(a) in the manuscript). Figure S2: amplicon bands generated from the genomic DNA of N. fowleri using the specific primers. Naegle: N. fowleri and M: 100 bp marker (uncropped image of gel of Figure 3(b) in the manuscript). [file 5514520.f1.docx]

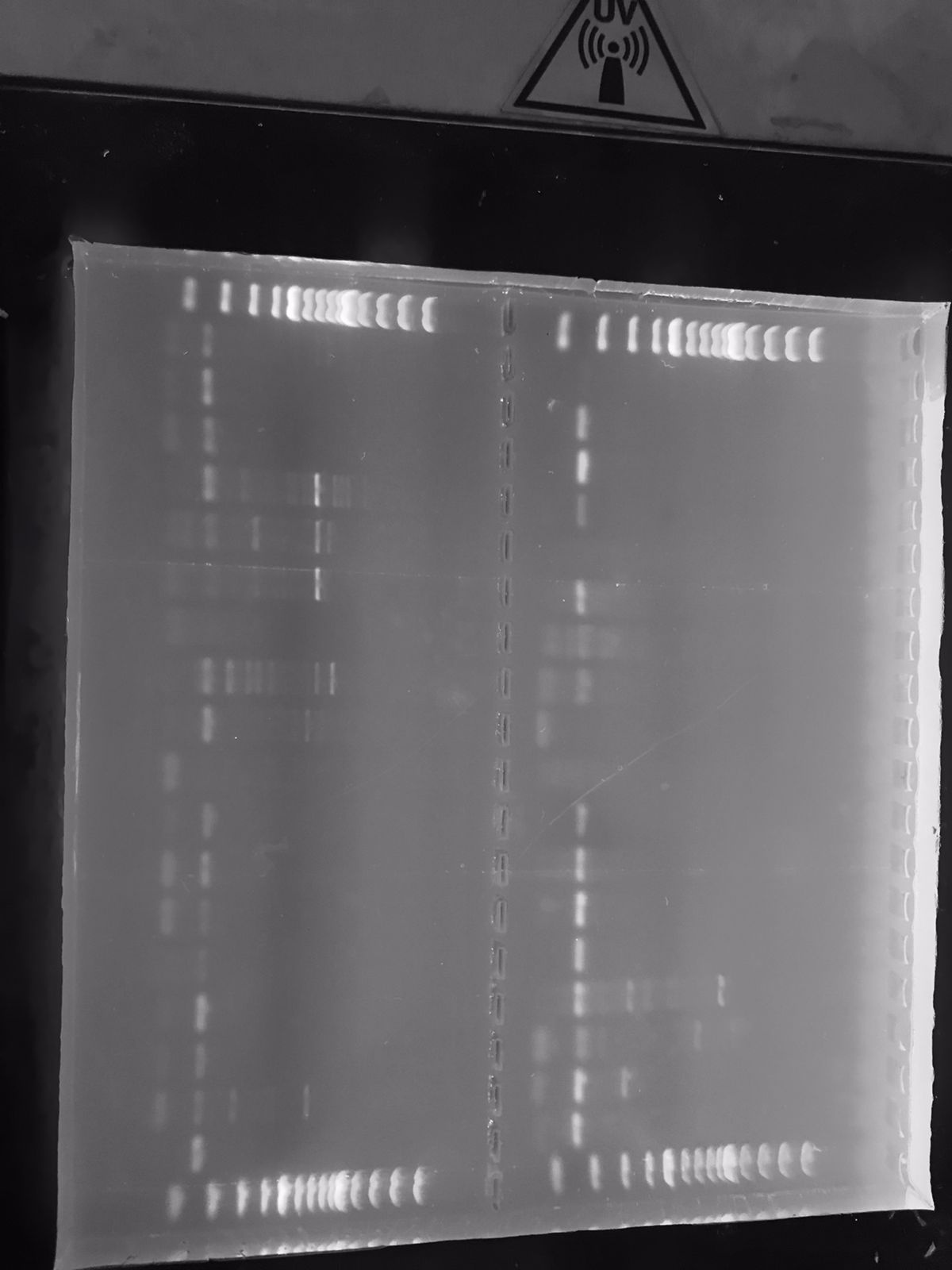


Figure S1:  Amplicon bands generated from the genomic DNA of *N. fowleri* using the specific primers. ITS-1 and M : 100 bp marker (Uncropped Image of Gel of Figure 3A in the manuscript)


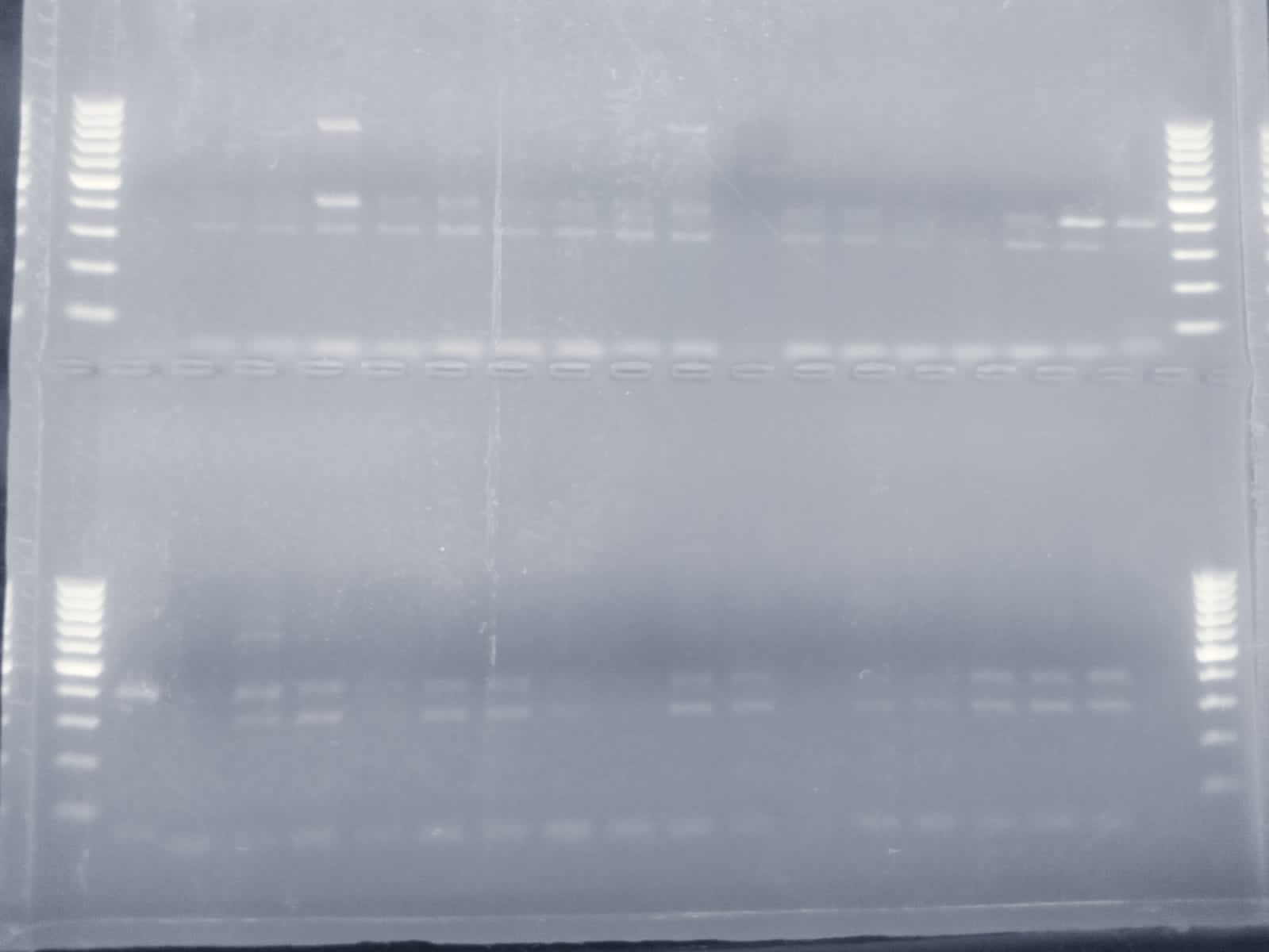


Figure S2:  Amplicon bands generated from the genomic DNA of *N. fowleri* using the specific primers. Naegle: *N. fowleri* and M: 100 bp marker (Uncropped Image of Gel of Figure 3B in the manuscript)
